# Genetic architecture of resistance to Septoria tritici blotch in European wheat

**DOI:** 10.1186/1471-2164-14-858

**Published:** 2013-12-05

**Authors:** Thomas Miedaner, Yusheng Zhao, Manje Gowda, C Friedrich H Longin, Viktor Korzun, Erhard Ebmeyer, Ebrahim Kazman, Jochen C Reif

**Affiliations:** State Plant Breeding Institute, University of Hohenheim, 70593 Stuttgart, Germany; Department of Cytogenetics and Genome Analysis, Leibniz Institute of Plant Genetics and Crop Plant Research (IPK), Gatersleben, Germany; KWS LOCHOW GMBH, 29296 Bergen, Germany; Lantmännen SW Seed Hadmersleben GmbH, 39398 Hadmersleben, Germany

**Keywords:** Association mapping, Genomic selection, Hybrid wheat, *Mycosphaerella graminicola*, *Septoria tritici*

## Abstract

**Background:**

Septoria tritici blotch is an important leaf disease of European winter wheat. In our survey, we analyzed Septoria tritici blotch resistance in field trials with a large population of 1,055 elite hybrids and their 87 parental lines. Entries were fingerprinted with the 9 k SNP array. The accuracy of prediction of Septoria tritici blotch resistance achieved with different genome-wide mapping approaches was evaluated based on robust cross validation scenarios.

**Results:**

Septoria tritici blotch disease severities were normally distributed, with genotypic variation being significantly (*P <* 0.01) larger than zero. The cross validation study revealed an absence of large effect QTL for additive and dominance effects. Application of genomic selection approaches particularly designed to tackle complex agronomic traits allowed to double the accuracy of prediction of Septoria tritici blotch resistance compared to calculation methods suited to detect QTL with large effects.

**Conclusions:**

Our study revealed that Septoria tritici blotch resistance in European winter wheat is controlled by multiple loci with small effect size. This suggests that the currently achieved level of resistance in this collection is likely to be durable, as involvement of a high number of genes in a resistance trait reduces the risk of the resistance to be overcome by specific pathogen isolates or races.

**Electronic supplementary material:**

The online version of this article (doi:10.1186/1471-2164-14-858) contains supplementary material, which is available to authorized users.

## Background

Septoria tritici blotch disease caused by *Mycosphaerella graminicola* (anamorph *Septoria tritici*) has become one of the most devastating leaf diseases in Central European winter wheat (*Triticum aestivum* L.). The high input of fungicides in combating this disease has led to a high percentage of fungal strains showing resistance to strobilurines [1]. Moreover, the sensitivity to some azoles also was reduced substantially [2]. Breeding for Septoria tritici blotch resistance, therefore, seems to be the most effective and environmental friendly method of control.

European winter wheat has a broad genetic basis of resistance to Septoria tritici blotch. In the descriptive list of cultivars of the German Federal Plant Variety Office [3], for example, Septoria tritici blotch ratings range from 3–8 on a 1–9 scale (1 = fully resistant) are documented. Sometimes resistance is isolate-specific based on a single *Stb* gene (*Stb1-18*, [4–7]) that can be monitored in a detached-leaf test [8], while in other cases quantitative resistance at the adult-plant stage is seen. The latter is in general based on several quantitative trait loci (QTL) with small to moderate effects [9, 10] and prone to high genotype x environment interactions [11]. Many European wheat varieties combine both types of resistances, as is illustrated by the wide range of Septoria tritici blotch ratings in cultivars with individual *Stb* genes of which several are not effective any more in the field [12]. A QTL analysis using four bi-parental populations revealed a total of 26 QTL for Septoria tritici blotch resistance, with the phenotypic variance explained by the individual QTL ranging from 3 to 21% [13]. QTL analysis also revealed that Septoria tritici blotch resistance was negatively correlated with plant height. As consequence, a significant effect of the plant height-controlling *Rht-D1* gene on Septoria tritici blotch resistance has been observed [14]. Negative associations between Septoria tritici blotch resistance and plant height as well as heading date were also detected in populations where *Rht-D1* was not segregating [14, 15]. Thus, tall and late wheat genotypes in general were found less prone to Septoria tritici blotch infection than short and early ones. The complex inheritance of Septoria tritici blotch resistance in European wheat has also been confirmed in a very recent study reporting 27 meta-QTL detected across seven bi-parental wheat populations (Goudemand, pers. commun.).

Despite success in detecting genomic regions involved in quantitative disease resistance, the underlying complex genetic architecture often prevents marker-assisted selection with high accuracy [16]. As an alternative concept, genomic selection has been proposed [17]. In genomic selection, effects are estimated for many markers based on large populations genotyped using high-density marker panels. First experimental results on Fusarium head blight resistance in wheat clearly suggested that genomic selection is very promising to improve breeding for quantitative disease resistances [18].

The current study relies on a diverse population of 1,055 European wheat hybrids and their 87 parental lines that were evaluated for adult-plant resistance to Septoria tritici blotch disease as well as fingerprinted with a 9 k SNP array. We applied one targeted association mapping and two genome-wide prediction approaches coupled with cross validations. Our objectives were to (1) study the potential to predict Septoria tritici blotch resistance in wheat and (2) evaluate the importance of additive, dominance and epistatic effects for Septoria tritici blotch resistance.

## Methods

### Plant material and field experiments

Our study was based on 1,055 elite wheat (*Triticum aestivum* L.) hybrids and their 87 parental lines (Additional file 1: Figure S1). The hybrids were derived by crossing 72 female and 15 male lines using chemical hybridization agents (Additional file 1: Figure S1). A detailed characterization of the population structure and extent of linkage disequilibrium of the parental lines have been discussed in our companion paper [19].

The in total 1,142 genotypes were evaluated for Septoria tritici blotch in unreplicated field trials at two locations in Schleswig-Holstein/Germany, Cecilienkoog (54.35 N, 8.55 E, at sea level) and Harzhof near Eckernförde (54.24 N, 9.51 E, 24 m above sea level), in the year 2012. The experimental design was an alpha design where replication and location effects were confounded. Sowing density ranged from 230 to 250 grains m^-2^ and plot size ranged from 0.56 to 1.5 m^2^.

In Cecilienkoog, targeted inoculation with a mixture of isolates was performed by spraying a spore suspension with a concentration of 1 × 10^6^ spores/ml for one time when all genotypes had fully expanded flag leaves. In Harzhof, natural infection was followed. Septoria tritici blotch disease severity was visually scored plot wise as coverage of flag leaves with lesions bearing pycnidia on a scale from 1 (fully resistant) to 9 (fully susceptible). Data were recorded on 28^th^ of June in both environments. Heading date (in days since January 1^st^) and plant height (in cm) were evaluated in addition.

### Genotypic data

Genotyping was done with the wheat 9 k SNP array based on the Illumina Infinium assay (for details see [19]). We performed quality checks for the SNP markers excluding those with (1) rate of missing values above 5%, (2) rate of heterozygosity above 5%, (3) and markers with minor allele frequency smaller than 0.05. In total, 1,280 SNP markers were used for the analyses. Additionally, genes *Rht-B1* and *Rht-D1* were genotyped by two functional markers (Korzun, pers. commun.) in the whole population of parents. Out of the 72 female lines, 25 and 45 lines turned out to carry dwarfing alleles at loci *Rht-B1* and *Rht-D1*, respectively. In addition, two male parents had a dwarfing allele at locus *Rht-B1*.

### Phenotypic data analyses

We performed lattice analyses of variance [20]. The variances of the hybrids were further split into variance due to general combining ability effects and variance due to specific combining ability effects [21]. Significance of variance components was tested by model comparison with likelihood ratio tests in which halved *P* values were used as an approximation [22]. Heritability on an entry-mean basis was estimated as the ratio of the genotypic (*σ*^*2*^_*G*_) versus the phenotypic variance (*σ*^*2*^_*P*_), *i.e.*, *σ*^*2*^_*G*_*/σ*^*2*^_*P*_. The phenotypic variance *σ*^*2*^_*P*_ comprises *σ*^*2*^_*G*_ and the masking variances divided by the number of locations. In addition, we assumed fixed genetic effects and estimated the Best Linear Unbiased Estimates of the 1,142 genotypes.

### Genome-wide mapping

We specified the additive and dominance design matrices for the hybrids and their parental lines according to the F_∞_ metric [23]. Based on the single location values we performed association mapping scans for additive and dominance effects with correcting for population stratification with a kinship matrix estimated based on the marker data as outlined in detail elsewhere [24]. We contrasted this approach with a model not correcting for population stratification (Additional file 2: Figure S2). The significance of genome-wide association mapping scans was estimated based on a false discovery rate (FDR) of 0.1. In addition, a two-dimensional genome scan was performed and additive × additive, additive × dominance, dominance × additive, and dominance × dominance digenic epistatic effects were tested. The model included the detected main effect QTL as co-factors as well as the main and interaction effects of the marker pair under consideration [25]. To test for significance, we followed the suggestion of [26] and divided the alpha-level of 0.05 by the number of possible independent tests for pairwise interactions assuming an extent of linkage disequilibrium of up to 5 cM (*P < 1.25e-6*). All above outlined statistical analyses were performed using the software ASReml 3.0 [27]. We used a multiple linear regression with all significant markers to estimate the proportion of the explained phenotypic variation. SNP markers were ordered according to their P values.

Based on the adjusted entry means of the 1,142 genotypes, we applied Ridge Regression Best Linear Unbiased Prediction (RR-BLUP) [28] and BayesC*π*[29, 30] considering additive and dominance effects. Details of the implementation of the models have been described in [24]. All statistical procedures for the genomic selection approaches were executed using R [31].

### Cross validations for association mapping and genomic selection

We evaluated the accuracy of prediction of Septoria tritici blotch resistance by association mapping and the two genomic selection approaches using cross validations. Since population structure in factorial crosses strongly influences prediction accuracy, we used a cross validation strategy where training and validation sets were not related to each other via common parental lines. We sampled 100 times 400 hybrids derived from 10 male and 47 female parental lines as training set and estimated the additive and dominance effects. Hybrids based on the remaining parental lines formed the validation set in which predictions derived from the training set were tested for their accuracy. For every training set, we applied the genomic selection as well as the association mapping models outlined above. For the association mapping approach, we corrected for population stratification with a kinship matrix and identified significant marker-trait associations using a false discovery rate (FDR) of 0.1. We predicted the hybrid performance using the estimated marker effects. The implementation of RR-BLUP was based on estimates of the genetic variances and heritability of the full population. Prediction accuracy was estimated as Pearson’s correlation coefficient between the observed and the predicted hybrid performance dividing by the square root of the heritability on an entry-mean basis. We used the heritability on an entry-mean basis estimated for the full population, because this estimate possesses a lower standard error compared to the heritability estimated in the training population.

## Results

**Figure 1:**
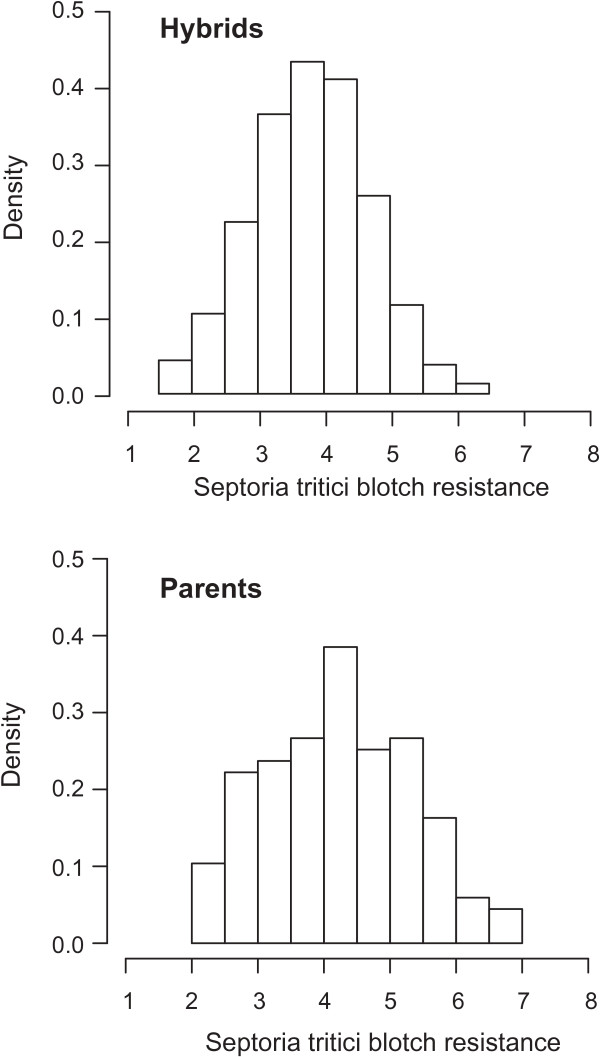
**Histograms of Septoria tritici blotch rating (1–9 scale, 1 = fully resistant) for 1,055 hybrids and their 87 parental lines across two environments after inoculation.**

Septoria tritici blotch disease severity revealed higher mean ratings for both lines and hybrids in Cecilienkoog than in Harzhof. The correlation between values estimated at both locations was significant (r = 0.32, *P <* 0.01). Among the parental lines, males showed a slightly lower Septoria tritici blotch severity than females (5.2 *vs*. 5.7). Across locations, Septoria tritici blotch severities amounted to on average 4.5 for parental lines and 4.0 for hybrids on the 1–9 scale. The distribution of the genotypic values of both material sets, lines and hybrids, followed approximately a normal distribution (Figure 1). Individual genotypes showed a similar and extremely large range for lines and hybrids. This resulted in large and significant genotypic variances for Septoria tritici blotch resistance (Table 1). Genotypic variation of the lines was about double as high compared to that of the hybrids. Partitioning of genotypic variance into general combining ability (GCA) and specific combining ability (SCA) variances resulted in a somewhat larger GCA of the females compared to the males (0.21 vs. 0.17, respectively, *P <* 0.001). Variance of SCA effects was not significantly larger than zero (0.02). Heritability estimates were high for the lines and moderate for the hybrids.

**Table 1:** **Means and ranges of Septoria tritici blotch resistance scores for 1,055 wheat hybrids and their 87 parental lines evaluated in field trials at two locations separately and across locations**

Source of variation	Cecilienkoog	Harzhof
*Means [Ranges]:*		
Lines	5.6 [2.0-8.0]	3.3 [1.0-6.0]
Hybrids	5.0 [2.0-8.0]	3.0 [1.0-6.0]
	Both locations	
*Means [Ranges]:*		
Lines	4.5 [2.0-7.0]	
Hybrids	4.0 [2.0-6.5]	
*Variances:*		
σ^2^_Lines_	0.83***	
σ^2^_Hybrids_	0.39***	
σ^2^_e_	0.72	
*Heritability*_*Lines*_	0.70	
*Heritability*_*Hybrids*_	0.52	

Septoria tritici blotch resistance was scored from 1 (fully resistant) to 9 (completely susceptible).

*** Significantly different from zero at 0.001 level of probability.

We observed a moderate, yet significant (r = 0.42, *P <* 0.001) correlation between plant height and Septoria tritici blotch severity (Additional file 3: Figure S3). The effects of dwarfing alleles *Rht-B1b* and *Rht-D1b* were significantly different from zero for the comparison-wise but not for the family-wise error rate and explained 4% and 1.2% of the total phenotypic variance for Septoria tritici blotch resistance, respectively. Heading time and Septoria tritici blotch severity were not significantly correlated (r = −0.03 for the hybrids).

In a genome-wide association mapping scan we detected eight SNPs (Table 2) significantly associated with genetic variation for Septoria tritici blotch resistance. Four of the related SNPs were not mapped. Highest frequency of occurrence was found for a SNP on chromosome 5B. The proportion of explained phenotypic variance ranged from 0.3% to 6.2%.

**Table 2:** **SNP markers detected in the association mapping scan underlying Septoria tritici blotch resistance, the proportion of explained phenotypic variance (R** ^**2**^ **), and the frequency of occurrence in the different cross validation runs**

Marker	Marker position (chromosome, centi Morgan)	***P*** value	R^2^	Frequency of occurrence (%)
we00819	1B (18)	4.82e-05	0.30	34
we01050	Not mapped	8.46e-06	2.76	61
we02521	6A (14.2)	5.49e-04	5.97	2
we03018	5B (53)	5.24e-04	6.21	63
we03636	2B (74.2)	2.93e-06	3.31	31
we04683	Not mapped	1.45e-04	4.62	52
we05375	Not mapped	4.14e-04	2.36	44
we06122	Not mapped	9.31e-05	0.33	16

**Figure 2:**
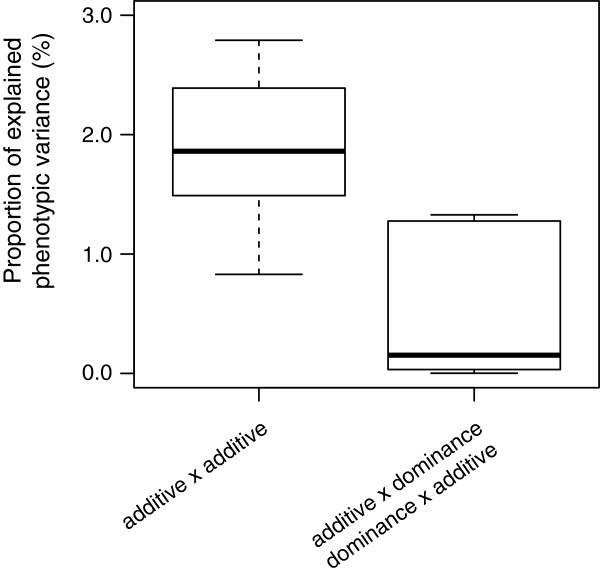
**Box-Whisker plots of the proportion of explained phenotypic variation for significant digenic epistatic effects.**

We performed a full two-dimensional scan for epistatic effects and observed a total of 40 significant digenic epistatic effects. The distribution of the *P* values revealed that among the four types of digenic epistatic effects, additive × additive interactions were the most prevailing ones (Figure 2, Additional file 4: Figure S4). In total, 31 additive x additive interactions were significantly different from zero, explaining on average 1.8% of phenotypic variation and 2.8% at maximum. Five additive x dominant interactions explained on average 0.79% (maximum 1.33%) and four dominant x additive interactions explained 0.24% (maximum 0.67%).

**Figure 3:**
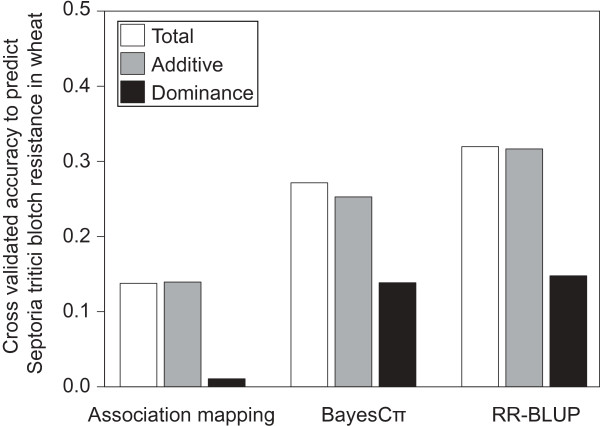
**Cross-validated accuracy to predict Septoria tritici blotch resistance in wheat based on association mapping and two genomic selection methods BayesCπ and RR-BLUP.**

Applying a robust cross validation strategy revealed that Septoria tritici blotch resistance can be predicted with an accuracy of 0.14 based on significant SNPs of the association mapping scans (Figure 3). Using only SNPs with significant additive effects did not lead to a reduction in the accuracy of prediction. By contrast, the two genomic selection approaches led to accuracies to predict Septoria tritici blotch resistance of 0.28 and 0.32, being more than double as high than the ones obtained with the association mapping approach. BayesCπ and RR-BLUP showed only small differences with a somewhat higher accuracy in RR-BLUP. Again, a combined prediction considering additive and dominance effects was only slightly superior to predicting Septoria tritici blotch resistance based on additive effects only.

## Discussion

Septoria tritici blotch in wheat has gained much attention in Europe in the last years. This is reflected by the substantial resources that were invested in programs for the selection for improved Septoria tritici blotch resistance. Inheritance of Septoria tritici blotch resistance is complex and QTL studies revealed only small effects of individual QTL. Among four populations, for example, 5 to 9 QTL per population were detected and the phenotypic variance explained by all QTL together dropped to 24% to 40% after five-fold cross validation [13]. Genomic selection has been proposed for the prediction of such complex traits that are controlled by multiple gene loci with small effects [17]. Therefore, we investigated in this survey the potential of genomic selection for Septoria tritici blotch resistance and contrasted the outcomes with those of association mapping.

### High disease severity resulted in good genetic differentiation

Artificial and natural infection led to a broad range of Septoria tritici blotch severity scores among lines and hybrids, illustrating the occurrence of resistance in the population (Table 1). Mean disease severity caused by natural infection was lower than after artificial inoculation. Nevertheless, resistance scores of genotypes seen with natural and with artificial infection were significantly (r = 0.32, *P <* 0.01) correlated. Similarly, Schilly et al. [11] had reported even higher correlations between artificial and natural infections. Accordingly, genotypic variances across locations were significantly (*P <* 0.01) different from zero for both lines and hybrids. Because natural *S. tritici* populations are highly variable in their race composition and most probably differed from the isolates used for inoculation, we can assume that we assessed broad-based adult-plant resistances in this study.

### Morphological resistance to Septoria tritici blotch

Septoria tritici blotch resistance is associated with increased plant height (Additional file 3: Figure S3). 18% of the phenotypic variance of Septoria tritici blotch resistance can most likely be attributed to increased plant height as calculated from the coefficient of correlation. This morphological resistance is most likely due to the mode of spread of pycnidiospores, the main source of inoculum in spring and summer. Pycnidiospores are splash dispersed and, thus, tall plants have a higher probability of escape infection than short ones [32].

On the level of genes, this morphological resistance was reflected in previous reports by the co-localization of QTL for Septoria tritici blotch resistance and with dwarfing gene loci [15, 33] as well as further plant height QTL [9]. In our study, the two dwarfing alleles, *Rht-B1b* and *Rht-D1b*, were well represented in the female lines. In addition, two male lines exhibited the *Rht-B1b* allele. The combined effect of the two dwarfing genes on Septoria tritici blotch severity amounted to 5%, which was not high enough to reach the significance threshold in the association mapping scan after correcting for multiple testing. This finding is surprising with regard to the large size of the mapping population. We thus investigated whether the population structure was associated with plant height, which could have caused a low power of QTL detection, but did not observe association between the distance matrix of the inbred lines and plant height. An alternative explanation is that in hybrids, which comprised the largest set of genotypes in our mapping population, the two dwarfing genes are mostly present at the heterozygous state. Combined with the observation that the dwarfing alleles exhibited only partial dominance (Zhao, pers. commun.), this might at least partially explain why the dwarfing genes did not show association with Septoria tritici blotch resistance at a genome wide level.

### QTL for septoria tritici blotch resistance revealed by association mapping

Eight SNPs were significantly associated with Septoria tritici blotch resistance (Table 2) with a SNP on chromosome 5B having the largest effect and frequency of occurrence (Table 2)*.* On this chromosome, the race-specific resistance gene *Stb1* is located [4]. Absence of functional markers for *Stb1*, however, prevents a further study in detail. Moreover, the source of the *Stb1* gene is a Bulgarian landrace, which has to our knowledge not yet been introgressed into Central European elite wheat lines. Two QTL from History/Rubens (34 cM, 68 cM) and one QTL from Arina/Forno (44 cM) have been also located in a similar region in a previous study on chromosome 5B [15]. In History/Rubens the QTL at 68 cM on chromosome 5B was additionally effective for Fusarium head blight resistance, thus, representing a broad-spectrum resistance QTL [14]. In accordance to our results, Eriksen et al. [9] detected a QTL on chromosome 2B, but the localization cannot be compared due to the use of a different type of markers.

### Relevance of intra- and interlocus interaction effects

The analysis of variance revealed a high importance of general combination ability effects as would be expected for quantitative pathogen resistances [34]. Variance of specific combining ability effects reflecting the variance of dominance effects [35] was not significantly different from zero. Consequently, the phenotypic data analysis suggested absence of net dominance effects. This finding is in accordance with the very low prediction accuracies in the association mapping approach observed when exploiting exclusively dominance effects, but in contrast to the positive prediction accuracies in the two genomic selection approaches (Figure 3). Interestingly, exploiting both additive and dominance effects in the prediction model led only to a marginal improvement as compared to using additive effects exclusively. This can partially be explained by the fact that dominance effects can be compensated by additive effects due to the non-orthogonal decomposition of the genotypic value applying the F_∞_ metric. A further reason for the low benefit to exploit besides additive also dominance effects is the large proportion of unexplained variation entering the prediction model when dominance effects are included [24].

We tested for the presence of epistatic effects among loci by a full two-dimensional scan. As expected for selfing species such as wheat [36], additive x additive effects are prevailing (Figure 3). In contrast, interaction effects involving dominance effects were less important. Across the different types of epistasis, the effect sizes of particular epistatic effects were small (Figure 2). Despite this, the huge number of potential interactions among loci in the genome can result in substantial cumulative epistatic effects (e.g., [37]).

A thorough evaluation of the potential to increase the prediction accuracy in genome-wide mapping approaches through the exploitation of epistasis requires cross validation studies. We have not performed these analyses, due to the high computational demand for two-dimensional scans of epistasis. There exists a strong demand to develop computational efficient methods to tackle epistatic interactions in genome-wide mapping studies based on high-density SNP arrays. Moreover, further research is needed to investigate methods suited to scale the epistatic variance components relative to the variance of the main effects.

### Doubling the prediction accuracy through genomic selection

Both applied genomic selection procedures had a considerably higher accuracy to predict Septoria tritici blotch resistance than the association mapping approach (Figure 3). Consequently, our findings clearly point to the need to tackle complex traits such as Septoria tritici blotch resistance with the appropriated biometrical models. A previous study on Fusarium head blight resistance, which presumably has a similar genetic architecture as of Septoria tritici blotch resistance [38], yielded a substantially higher prediction accuracy of 0.61 despite a lower population size [18]. This higher prediction accuracy might be explained by differences in the genetic composition of the lines of both studies. Using SSR markers, we have selected a set of parental lines with maximized allelic diversity out of several hundred elite lines. Such a strategy strongly decreases the average degree of relatedness in the population under consideration. Therefore, genomic selection mainly exploits linkage disequilibrium between SNPs and QTL [39]. It should also be noted that we followed a conservative cross validation procedure to allow generalization of our results, *i.e.* training and validation sets did not share any parents. If the information for the female or male parents is additionally implemented in the model, the accuracy rises to 0.6 (male parents) or 0.87 (female parents).

## Conclusion

For our data set, we observed a slight superiority of RR-BLUP compared to BayesCπ. While RR-BLUP approximates the infinitesimal model, BayesCπ relies on the assumption that particular loci contributed stronger to the phenotypic variance than others [24]. Consequently, our finding can be interpreted as an indicator that Septoria tritici blotch resistance is controlled by many loci with a small effect size each. Given the accuracy of genomic prediction in this population, phenotypic selection of quantitative Septoria tritici blotch resistance should be continued. The results give a good prospect for the durability of Septoria tritici blotch resistance present in the underlying population, as a high number of genes responsible for one resistance trait is reducing the risk of the resistance being overcome by a specific isolate or race.

## Availability of data

The data is available from the Dryad Digital Repository: doi:10.5061/dryad.461nc.

## Electronic supplementary material

Additional file 1: Figure S1: Crossing scheme between the 72 female and 15 male wheat lines. Open boxes indicate presence and filled boxes indicate absence of a particular cross. (EPS 807 KB)

Additional file 2: Figure S2: Quantile-quantile plots for association mapping based on the combined population of hybrids and lines using two different biometrical approaches, (1) without correction for population structure, and (2) correcting for population structure with a kinship matrix. (EPS 830 KB)

Additional file 3: Figure S3: Association of Septoria tritici blotch rating (1–9, 1 = fully resistant) and plant height for 1,055 hybrids and 135 lines, r = coefficient of correlation, *** = *** Significantly different from zero at 0.001 level of probability. (EPS 809 KB)

Additional file 4: Figure S4: Distribution of *P* values for 4 different types of digenic epistatic effects. (EPS 1 MB)
